# Effect of the thumbtack needle on gastrointestinal function recovery after laparoscopic radical gastrectomy for gastric cancer with the concept of enhanced recovery after surgery: a randomized controlled trial

**DOI:** 10.3389/fsurg.2025.1612766

**Published:** 2025-09-18

**Authors:** Shuai Guo, Xiang-Ping Lin, Xiang-Ren Jin, Kang-Xiu Tuo, Pei Li, Wei-Wei Yang, Qian Wang

**Affiliations:** ^1^ Department of Pharmacy, The Affiliated Hospital of Guizhou Medical University, Guiyang, Guizhou, China; ^2^ Department of Gastroenterology, The Affiliated Hospital of Guizhou Medical University, Guiyang, Guizhou, China

**Keywords:** thumbtack needle, gastric cancer, GI function recovery, laparoscopic radical gastrectomy, ERAS

## Abstract

**Objectives:**

Postoperative gastrointestinal (GI) dysfunction is a common complication in patients undergoing gastric cancer surgery. This study aimed to evaluate the effect of thumbtack needle therapy on GI function recovery after laparoscopic radical gastrectomy.

**Methods:**

Participants were randomly assigned to either the treatment or control group. Both groups received perioperative enhanced recovery after surgery management. Participants in the treatment group received thumbtack needle therapy at bilateral Neiguan (PC6), Zusanli (ST36), Shangjuxu (ST37), Hegu (LI4), and Sanyinjiao (SP6). Primary outcomes included the time to bowel sound recovery and time to first flatus (all measured in hours). Secondary outcomes included the time to first defecation, time to removal of the nasogastric tube and intra-abdominal drains (all measured in hours), postoperative pain scores, nausea and vomiting scores, abdominal distension scores (all measured in points), length of hospital stay (days), incidence of complications (%), safety evaluation, and overall response rate (%).

**Results:**

A total of 103 participants were screened, and 80 were enrolled (40 per group). Baseline characteristics were similar between groups. Compared with the control group, the treatment group showed significantly shorter times to bowel sound recovery (difference, −4.0 h, 95% CI: −7.0 to −1.0, *P* = 0.010), first flatus (−11.0 h, 95% CI: −19.0 to −2.0, *P* = 0.017), first defecation (−8.0 h, 95% CI: −16.0 to −1.0, *P* = 0.026), nasogastric tube removal (−12.0 h, 95% CI: −27.0 to −2.0 *P* = 0.023), and intra-abdominal drain removal (−10.0 h, 95% CI: −21.0 to −1.0, *P* = 0.038). Pain scores were significantly lower in the treatment group on postoperative day (POD) 1 (−1, 95% CI: −1 to 0, *P* = 0.031), POD 2 (−1, 95% CI: −2 to −1, *P* < 0.001), and POD 3 (−1, 95% CI: −2 to 0, *P* < 0.001). Similar improvements were observed in nausea, vomiting, and abdominal distension scores on POD 1–3 (all showing a median difference of −1, all *P* < 0.05). The treatment group also had a significantly shorter hospital stay (difference, −1.7 days, 95% CI: −3.0 to −0.3, *P* *=* 0.015). There was no significant difference in the incidence of postoperative complications (difference, −5.0%, 95% CI: −18.6 to 8.0, *P* *=* 0.396), and no adverse reactions occurred in the treatment group. The overall response rate was significantly higher in the treatment group (difference, 17.5%, 95% CI: 0.18–34.0, *P* *=* 0.046).

**Conclusion:**

Thumbtack needle therapy at bilateral Neiguan (PC6), Zusanli (ST36), Shangjuxu (ST37), Hegu (LI4), and Sanyinjiao (SP6) is a safe and effective intervention that promotes early recovery of GI function after laparoscopic radical gastrectomy for gastric cancer.

**Clinical Trial Registration:**

http://www.chictr.org.cn, ChiCTR2400084712.

## 1 Introduction

In recent years, the incidence and mortality of gastric cancer have risen significantly. In 2020, approximately 1.089 million new cases and 769,000 deaths were reported globally, with gastric cancer ranking fifth in incidence and fourth in cancer-related mortality worldwide (1). Laparoscopic radical surgery for gastric cancer has become the most common surgical method for tumor resection. However, postoperative gastrointestinal dysfunction (PGD) remains an inevitable complication due to surgical trauma and the use of anesthetics (2). Clinically, PGD manifests as diminished or absent bowel sounds, involuntary flatulence or defecation, and symptoms such as abdominal distension, pain, nausea, and vomiting. If GI function is not promptly restored, complications such as delayed wound healing and severe abdominal distension may occur. Although postoperative paralytic (adynamic) ileus is a common transient functional disorder, it should be clearly distinguished from mechanical ileus, which is characterized by a physical obstruction and represents a more serious postoperative complication. In rare cases, prolonged paralytic ileus may lead to vascular compromise and further complications; however, it does not involve anatomic blockage (3).

The enhanced recovery after surgery (ERAS) concept has gained increasing prominence among clinicians in recent years. ERAS emphasizes the application of evidence-based perioperative strategies aimed at reducing surgical stress, minimizing complications, accelerating recovery, and shortening hospital stay (4–6). Within the ERAS framework, Western medicine approaches primarily focus on standardized care and symptomatic pharmacological support to facilitate recovery. Notably, acupuncture has been recognized in clinical guidelines as an effective non-pharmacologic intervention, and its application in managing PGD has gradually increased (7, 8). Postoperative acupuncture has been shown to regulate visceral function and provide significant clinical benefits (9).

The thumbtack needle technique, a form of superficial acupuncture, involves embedding a small needle under the skin at specific acupoints to provide continuous stimulation. This sustained input is believed to regulate the functions of meridians, internal organs, and Qi blood (i.e., microcirculatory and neuromodulatory dynamics) (10). Its therapeutic rationale in PGD is based on the modulation of cutaneous-visceral reflex pathways, which integrate somatic afferents with vagal and splanchnic efferents, enabling bidirectional communication between peripheral stimulation sites and visceral organs (11). From a modern biomedical perspective, surgical trauma and anesthesia contribute to PGD through multiple mechanisms. Anesthetics inhibit gastric motility by suppressing vagal activity and delaying gastric emptying, while surgical stress induces systemic inflammation, resulting in intestinal paralysis and impaired GI motility. These effects are further exacerbated by postoperative oxidative stress and mitochondrial dysfunction, which impair enteric nervous system (ENS) signaling and smooth muscle contractility (12–15). Therefore, effective therapeutic strategies should target vagal activation and ENS–smooth muscle coordination to restore integrated gastric and intestinal function through neuromodulation (16–18).

**Figure 1:**
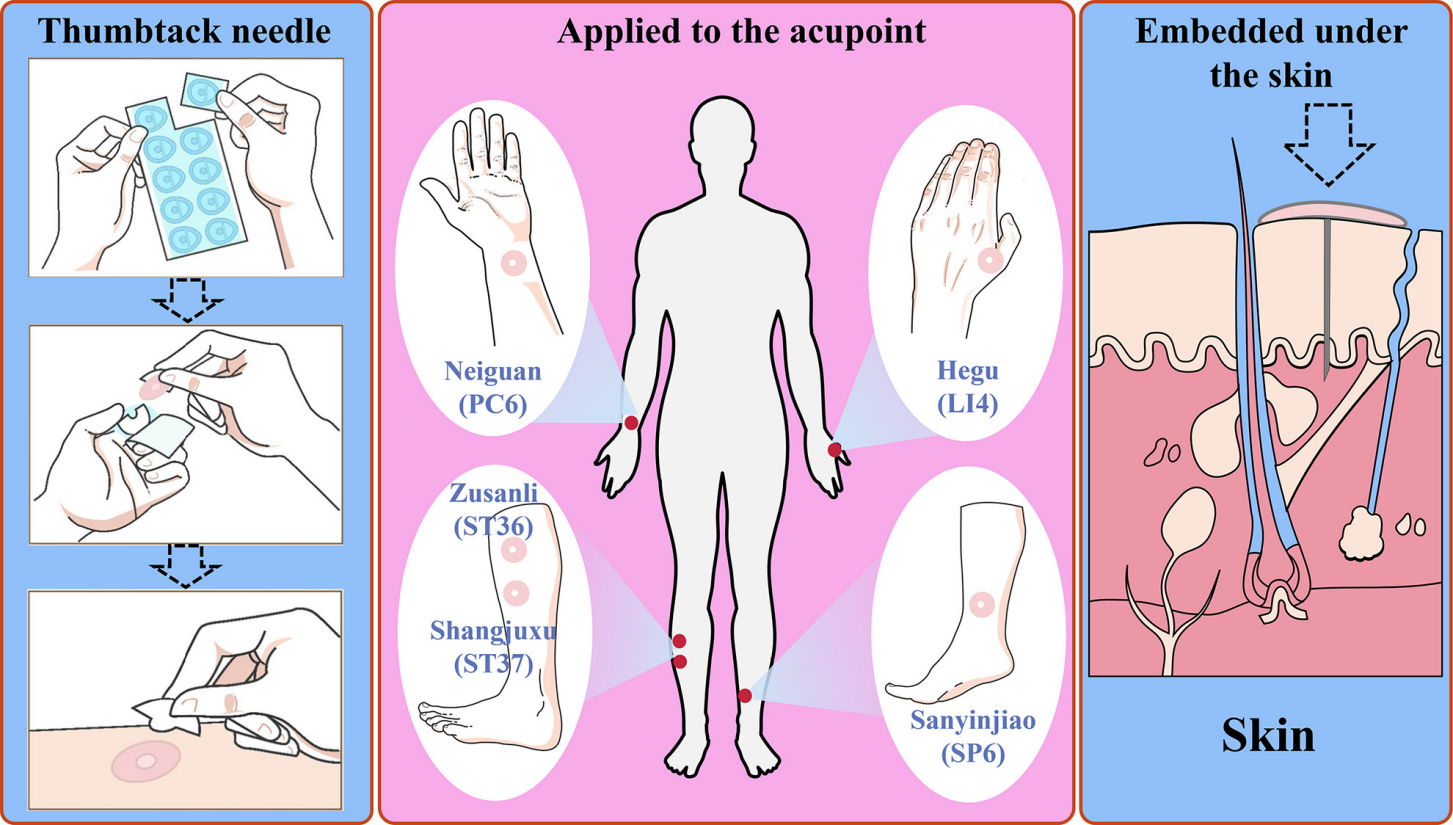
Schematic illustration of thumbtack needle therapy for improving GI function recovery through continuous stimulation of acupoints: Neiguan (PC6), Hegu (LI4), Zusanli (ST36), Shangjuxu (ST37), and Sanyinjiao (SP6).

In this study, thumbtack needles were bilaterally placed at Neiguan (PC6), Zusanli (ST36), Shangjuxu (ST37), Hegu (LI4), and Sanyinjiao (SP6) to provide continuous stimulation of key acupoints and promote GI recovery via cutaneous-visceral reflex pathways (Figure 1). This study aimed to evaluate whether thumbtack needle therapy can facilitate early recovery of GI function following laparoscopic radical gastrectomy for gastric cancer and assess its safety.

## 2 Materials and methods

This was a prospective, single-center, randomized controlled, and non-blinded trial conducted in the Department of Gastroenterology at the Affiliated Hospital of Guizhou Medical University. The trial was approved by the Ethics Committee of the Affiliated Hospital of Guizhou Medical University and was registered in the Chinese Clinical Trial Registry (ChiCTR2400084712). All eligible participants provided written informed consent prior to enrollment. The study followed the Consolidated Standards of Reporting Trials (CONSORT) reporting guideline.

Participants included gastric cancer patients who were scheduled to undergo laparoscopic surgery at the Affiliated Hospital of Guizhou Medical University from June 2024 to February 2025. The inclusion criteria were as follows: (1) age 18–70 years, regardless of gender; (2) histologically confirmed gastric cancer based on gastroscopy and pathological examination; (3) scheduled for laparoscopic radical gastrectomy; and (4) voluntary participation with signed informed consent. The exclusion criteria included (1) known metal allergy or significant needle phobia; (2) surgical incisions or scarring in the meridian areas corresponding to Neiguan (PC6), Zusanli (ST36), Shangjuxu (ST37), Hegu (LI4), or Sanyinjiao (SP6); (3) local skin infections at the aforementioned acupoints; (4) inability to comprehend or complete the visual analog scale (VAS), numeric rating scale (NRS), or gastrointestinal symptom rating scale (GSRS); (5) conversion to open surgery; (6) occurrence of serious adverse events (AEs); and (7) judged by investigators to be unsuitable for participation.

**Figure 2:**
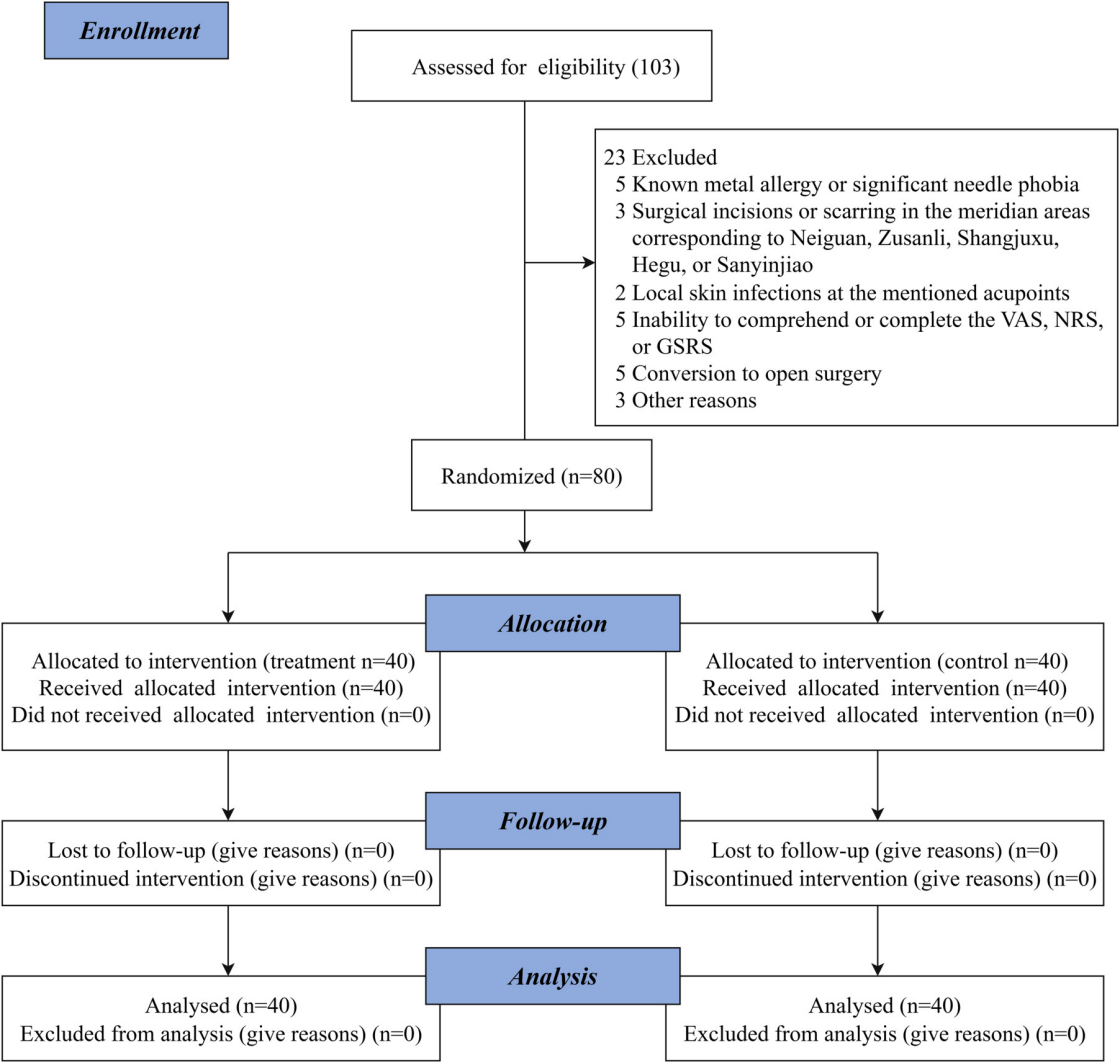
Patient enrollment flowchart.

The patient enrollment flowchart is presented in Figure 2.

### 2.1 Intervention

All patients underwent laparoscopic resection of gastric cancer. Specifically, all surgeries were performed using 3D laparoscopic techniques and included distal, proximal, or total gastrectomy depending on the tumor location and clinical staging. A standardized D2 lymphadenectomy combined with complete mesogastrium excision was performed in all cases.

#### 2.1.1 ERAS perioperative management

All participants received ERAS perioperative management (19–21):

1. Preoperative period

• Preoperative education: On the day of admission, patients were educated on the components and anticipated benefits of ERAS-based perioperative management to ensure their understanding and cooperation, as well as that of their families.

• Nutritional risk assessment: For patients with nutritional risk (NRS2002 ≥ 3 points), enteral or parenteral nutrition is provided based on whether the patient is fasting.

• Bowel preparation: Mechanical bowel preparation was not performed.

• Fasting: Patients were instructed to fast from solid food for 6 h and from clear liquids for 2 h before surgery.

• Perioperative prophylactic antithrombotic therapy: The Caprini thrombosis risk assessment scale was used to evaluate the risk of venous thromboembolism at admission.

• Prophylactic antibiotic use: Antibiotics were administered 30 min before surgery.

2. Intraoperative period

• Anesthesia management: General and intravenous combined anesthesia.

• Temperature management: Intraoperative body temperature was monitored. Warm distilled water was used for peritoneal lavage, and a warming blower was applied to maintain normothermia during surgery.

• Fluid management: No fluid restriction.

• Intra-abdominal drains management: Selectively placed based on the patient’s intraoperative surgical condition, and removed early, within 1–2 postoperative days.

• Nasogastric tube management: Inserted intraoperatively and removed within 24 h postoperatively if the anastomosis was deemed satisfactory.

• Urinary catheter management: Inserted intraoperatively and removed early, within 1–2 postoperative days.

3. Postoperative period

• Pain management: Adequate postoperative analgesia was provided using a multimodal analgesic strategy, including low-dose opioids combined with NSAIDs, along with patient-controlled intravenous analgesia.

• Prevention and treatment of nausea and vomiting: 5-HT₃ receptor antagonists were administered intravenously.

• Dietary guidance: Except in patients with impaired intestinal function, anastomotic leakage, bowel obstruction, or high risk of gastroparesis, we initiate orally ingested nutritional support within 24 h after surgery.

• Mobilization out of bed 24 h after surgery.

• Fluid management: A goal-directed fluid therapy approach, which aims to maintain appropriate tissue perfusion and organ function while avoiding both hypovolemia-related complications and volume overload.

#### 2.1.2 Acupuncture procedures

Patients in the treatment group received thumbtack needle therapy using disposable, sterile needles (*Φ*0.20 × 1.0 mm, Hwato, Suzhou Medical Appliance Factory, China), while those in the control group did not receive any acupuncture intervention. The selected bilateral acupoints were Neiguan (PC6), Zusanli (ST36), Shangjuxu (ST37), Hegu (LI4), and Sanyinjiao (SP6) (detailed acupoint locations are provided in Figure S1 and Table S1 in the Supplementary Material). Acupoint nomenclature and anatomical locations followed the National Standard of the People’s Republic of China Nomenclature and Location of Meridian Points (GB/T 12346-2021), established in 2021 (22).

Before application, the skin at the acupoint sites was disinfected using 75% alcohol. A disposable thumbtack needle was then applied to each acupoint with vertical pressure using the thumb or index finger, starting gently and increasing until a tingling sensation (deqi) was achieved. Each session involved stimulating each acupoint for 1 min, repeated every 4 h. The treatment was administered once daily, beginning 2 days prior to surgery and continuing until 3 days after surgery.

### 2.2 Outcomes

The primary outcomes were the time to bowel sound recovery and time to first flatus, both measured in hours.

The secondary outcomes included the time to first defecation, time to removal of nasogastric tube and intra-abdominal drains (measured in hours), postoperative pain score (assessed using the NRS), postoperative nausea and vomiting score (assessed using the VAS), postoperative abdominal distention score (assessed using the GSRS), postoperative hospital stay, incidence of postoperative complications, safety evaluation, and overall response rate. The overall response rate was assessed 72 h postoperatively, based on the Rome IV criteria. It was defined as the sum of patients achieving complete response, marked response, or moderate response. Postoperative pain, nausea and vomiting score, and abdominal distention scores were recorded at three time points: POD 1, POD 2, and POD 3. Patients self-evaluated their symptoms using the corresponding validated numerical scales.

Detailed definitions and assessment criteria for both primary and secondary outcomes are presented in Table S2 in the Supplementary Material.

### 2.3 Sample size

In this study, the expected difference between the treatment and control groups was estimated based on a previous study (23), which reported a postoperative time to first flatus of 32.03 ± 8.42 h in the acupuncture group vs. 38.03 ± 7.31 h in the control group. This yielded an expected mean difference of approximately 6 h, which was used as the basis for sample size calculation. Assuming a two-sided significance level of *α* = 0.05 and a power (1 − *β*) of 80%, the corresponding *Z* values for a bilateral test were *Z_α_*_/2_ = 1.96 and *Z_β_* = 0.84. The ratio between the treatment group and control group was set at 1:1. The sample size was calculated using the following formula: $n=(Z_{\alpha /2}+Z_{\beta }{)}^{2}\cdot (\sigma_{1}^{2}+\sigma_{2}^{2}{)}^{2}/\delta^{2}$. A sample size of 36 per group was calculated, and with an estimated 10% dropout rate, 40 patients were enrolled in each group (total *N* = 80).

### 2.4 Randomization and blinding

Eligible patients were randomly assigned to either the treatment group or control group in a 1:1 ratio using SPSS version 26.0 to generate the randomization sequence. The allocation results were placed sequentially into sealed, opaque envelopes, which were prepared and managed by the individual who generated the randomization sequence**.** An envelope was opened only after a participant met all inclusion and exclusion criteria and provided informed consent. Perioperative management was then conducted according to the group assignment indicated in the envelope.

The researcher who generated the randomization sequence was not involved in participant enrollment or group assignment. Furthermore, the individual responsible for generating and maintaining the randomization list was not involved in any other aspect of the study.

### 2.5 Statistical analysis

Statistical analyses were performed using SPSS 26.0 and R 4.1.0 software. Descriptive statistics were used to summarize the characteristics of patients in each group. For continuous variables, normality was assessed using the Shapiro–Wilk test. Data conforming to a normal distribution were presented as mean ± standard deviation (SD), and between-group comparisons were conducted using the independent samples *t*-test. Non-normally distributed data were reported as median [interquartile range (IQR)], and compared using the Mann–Whitney *U* test. The Hodges–Lehmann estimator was applied to compute the median differences and associated 95% confidence intervals (CIs). Categorical variables were compared using the chi-square (*χ*^2^) test or Fisher’s exact test, as appropriate. For variables with repeated measures and a normal distribution, analysis of variance (ANOVA) was used, followed by least significant difference (LSD) *post-hoc test*s for pairwise comparisons at different time points. For non-normally distributed repeated measures, the Scheirer–Ray–Hare test was applied. A two-sided *P* < 0.05 was considered statistically significant.

## 3 Results

### 3.1 Baseline patient characteristics

A total of 103 participants were screened for eligibility. Of these, 23 were excluded, and 80 participants were randomly assigned to either the treatment group (*n* = 40) or the control group (*n* = 40), resulting in a recruitment rate of 77.7%. The study population included 53 men (66.3%) and 27 women (33.7%). All randomized participants completed the trial, and there were no losses to follow-up (Figure 2).

Baseline demographic and clinical characteristics of the two groups are presented in Table 1. There were no statistically significant differences between groups in terms of age, sex, BMI, operation time, medical costs, TNM, surgical procedure, or preoperative symptom scores.

**Table 1:** Baseline characteristics of participants.

Variable	Participants, no. (%)		
	Treatment group	Control group	*P*-value
	(*n* = 40)	(*n* = 40)	
Age, mean ± SD, years	62.2 ± 8.7	61.9 ± 8.8	0.085
Sex, no. (%)			
Male	24 (60.0)	29 (72.5)	0.344
Female	16 (40.0)	11 (27.5)	
BMI, mean ± SD, kg·m^−2^	23.0 ± 4.4	21.3 ± 3.5	0.069
Operation time, mean ± SD, min	277.5 ± 40.6	273.7 ± 33.6	0.650
Medical costs, mean ± SD, CNY	55,961.2 ± 10,621.6	61,724.3 ± 16,361.6	0.065
Preoperative pain, median (IQR), NRS score^a^	4.0 (3.3, 5.0)	4.0 (3.0, 4.0)	0.113
Preoperative nausea and vomiting, median (IQR), VAS score^b^	3.0 (1.0, 4.0)	2.5 (1.0, 4.0)	0.603
Preoperative abdominal distention, median (IQR), GSRS score^c^	3.0 (2.0, 4.0)	3.0 (2.0, 4.0)	0.937
TNM, no. (%)			
I	16 (40.0%)	9 (22.5%)	0.273
II	10 (25.0%)	9 (22.5%)	
III	13 (32.5%)	21 (52.5%)	
IV	1 (2.5%)	1 (2.5%)	
Surgical procedure, no. (%)			
Proximal gastrectomy	2 (5.0%)	5 (12.5%)	0.215
Distal gastrectomy	35 (87.5%)	28 (70%)	
Total gastrectomy	3 (7.5%)	7 (17.5%)	

BMI, body mass index; CNY, Chinese yuan; TNM, tumor node metastasis.

^a^ The NRS has a range from 1 to 10, with higher scores indicating worse pain.

^b^ The VAS has a range from 1 to 10, with higher scores indicating worse nausea and vomiting.

^c^ The GSRS has a range from 1 to 7, with higher scores indicating worse abdominal distention.

### 3.2 Primary outcomes

Bowel sounds serve as a direct indicator of intestinal peristalsis, and their postoperative recovery reflects the gradual resolution of postoperative ileus and the restoration of GI function. First flatus provides objective evidence of resumed intestinal transit, suggesting that mechanical obstruction has resolved and preliminary digestive function has returned. These two primary outcomes together offer a comprehensive assessment of GI function recovery.

**Figure 3:**
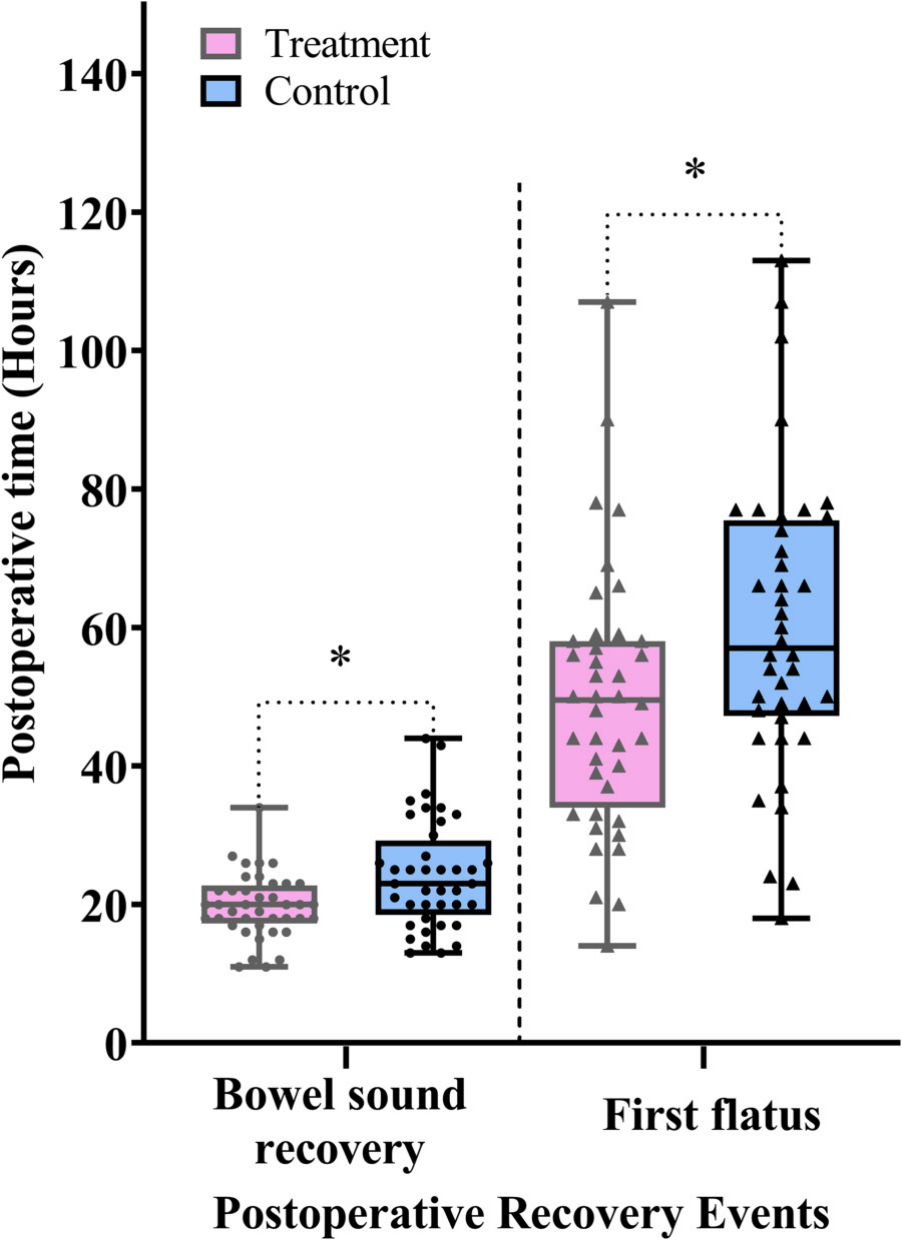
Comparison of the postoperative time to bowel sound recovery and time to first flatus between the treatment and control groups. Data are expressed as the median (IQR) (*n* = 40/group). Statistical significance was defined as *P < 0.025, adjusted using the Bonferroni correction for the two primary outcomes.

As shown in Figure 3 and Table 2, the treatment group experienced significantly shorter times to bowel sound recovery and first flatus compared with the control group (difference, −4.0 h, 95% CI: −7.0 to −1.0, *P* *=* 0.010; difference, −11.0 h, 95% CI: −19.0 to −2.0, *P* *=* 0.017). These findings suggest that thumbtack needle therapy may effectively accelerate the recovery of GI function following laparoscopic gastrectomy.

**Table 2:** Primary and secondary outcomes.

Outcomes	Treatment group	Control group	Difference	*P*-value
	(*n* = 40)	(*n* = 40)	(95% CI)	
Time to bowel sound recovery, median (IQR), h	20.0 (17.3, 22.8)	23.0 (18.5, 29.3)	−4.0 (−7.0 to −1.0)	0.010^a^
Time to first flatus, median (IQR), h	49.5 (34.0, 58.0)	57.0 (47.3, 75.5)	−11.0 (−19.0 to −2.0)	0.017^a^
Time to first defecation, median (IQR), h	70.0 (62.3, 77.8)	78.0 (67.0, 88.8)	−8.0 (−16.0 to −1.0)	0.026
Time to nasogastric tube removal, median (IQR), h	59.0 (40.0, 75.5)	72.0 (58.5, 99.0)	−12.0 (−27.0 to −2.0)	0.023
Time to intra-abdominal drains removal, median (IQR), h	117.0 (111.0, 138.5)	132.5 (117.8, 143.0)	−10.0 (−21.0 to −1.0)	0.038
Postoperative pain score, median (IQR), NRS score				
POD 1	7 (6.0, 8.0)	8 (7.0, 8.0)	−1.0 (−1.0 to 0.0)	0.031
POD 2	5 (4.0, 6.0)	7 (6.0, 7.0)	−2.0 (−2.0 to 1.0)	<0.001
POD 3	3 (2.0, 4.0)	4 (3.0, 5.0)	−1.0 (−2.0 to 0.0)	0.001
Postoperative nausea and vomiting score, median (IQR), VAS score				
POD 1	4 (2.3, 5.0)	5 (3.0, 6.0)	−1.0 (−2.0 to 0.0)	0.034
POD 2	2 (1.0, 3.0)	3 (2.0, 4.0)	−1.0 (−2.0 to −1.0)	0.001
POD 3	1 (0.0, 1.8)	2 (1.0, 3.0)	−1.0 (−2.0 to −1.0)	<0.001
Postoperative abdominal distention score, median (IQR), GSRS score				
POD 1	3.5 (3.0, 4.0)	4 (3.0, 5.0)	−1.0 (−1.0 to 0.0)	0.047
POD 2	2.5 (1.0, 3.0)	3 (2.0, 4.0)	1.0 (−1.0 to 0.0)	0.028
POD 3	1 (1.0, 2.8)	3 (2.0, 3.0)	−1.0 (−1.0 to 0.0)	0.003
Postoperative hospital stays, mean ± SD, days	9.1 ± 1.9	10.7 ± 3.8	−1.7 (−3.0 to −0.3)	0.015
Postoperative complications, no. (%)	2 (5.0)	4 (10.0)	−5.0 (−18.6 to 8.0)	0.396
Overall response rate^b^, no. (%)	36 (90.0)	29 (72.5)	17.5 (0.2 to 34.0)	0.046

^a^ *P* < 0.025 was considered statistically significant, adjusted for multiple comparisons using the Bonferroni correction.

^b^ Overall response rate was defined as the sum of complete response, marked response, and moderate response, based on the Rome IV criteria.

### 3.3 Secondary outcomes

#### 3.3.1 The time to first defecation and removal of nasogastric tube and intra-abdominal drains

The first defecation marks the recovery of colonic function. Removal of the nasogastric tube enables patients to gradually resume oral intake, which in turn stimulates GI hormone secretion and establishes a positive feedback loop that promotes GI functional recovery. Following the removal of the intra-abdominal drains, early ambulation becomes feasible, further stimulating intestinal peristalsis and accelerating postoperative GI recovery.

**Figure 4:**
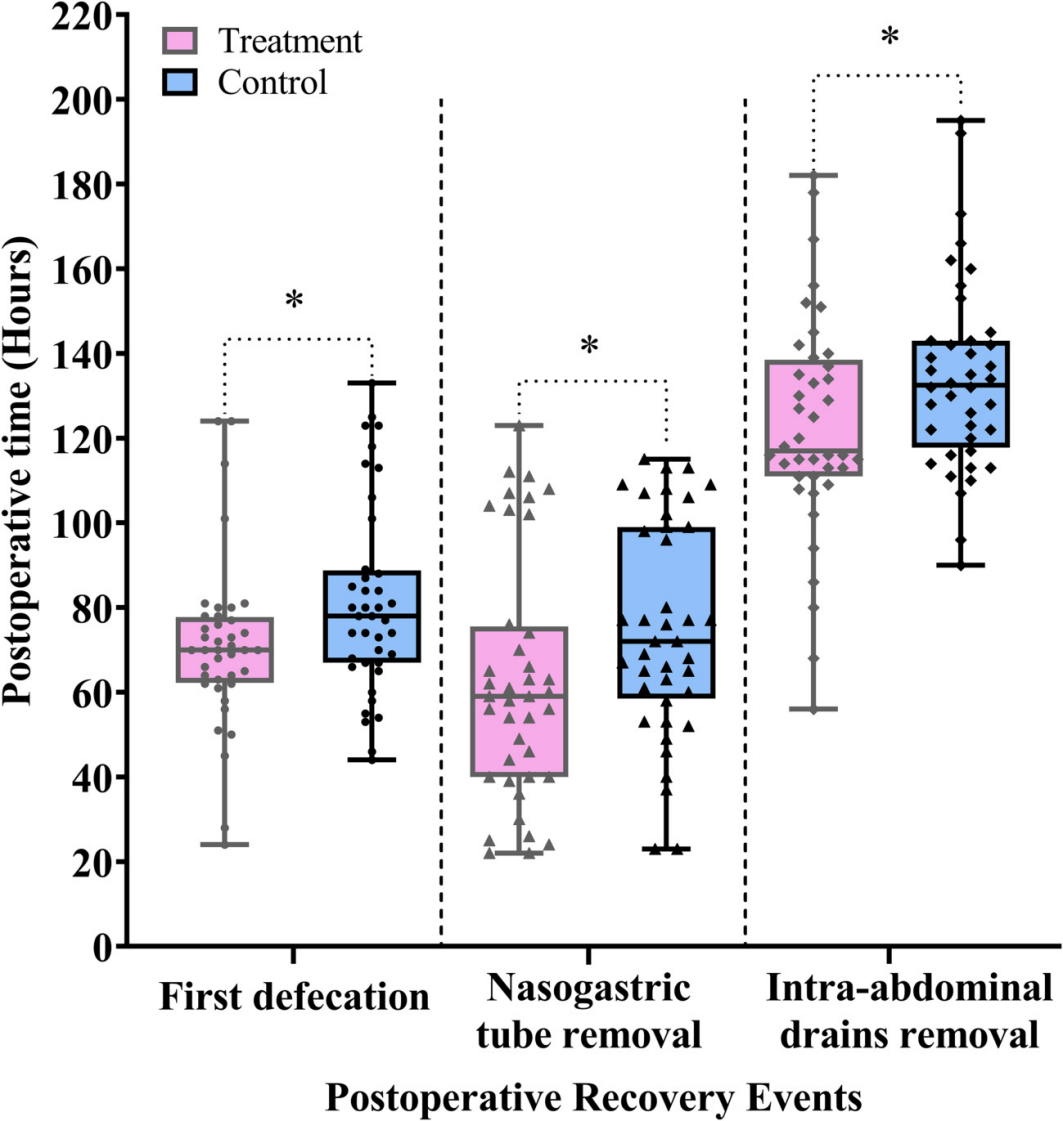
Comparison of the postoperative time to first defecation and time to removal of nasogastric tube and intra-abdominal drains between the treatment and control groups. Data are expressed as the median (IQR) (*n* = 40/group). Statistical significance was defined as * = *P* < 0.05.

As shown in Figure 4 and Table 2, the treatment group demonstrated significantly shorter times to first defecation, nasogastric tube removal, and intra-abdominal drains removal compared with the control group (difference, −8.0 h, 95% CI: −16.0 to −1.0, *P* *=* 0.026; difference, −12.0 h, 95% CI: −27.0 to −2.0, *P* *=* 0.023; difference, −10.0 h, 95% CI: −21.0 to −1.0, *P* *=* 0.038).

#### 3.3.2 Postoperative pain score

Postoperative pain is a key parameter in evaluating the effectiveness of perioperative management. In laparoscopic surgery, the use of carbon dioxide to establish pneumoperitoneum may irritate the peritoneal nerves and contribute to postoperative pain. In this study, pain levels were assessed using the NRS.

**Figure 5:**
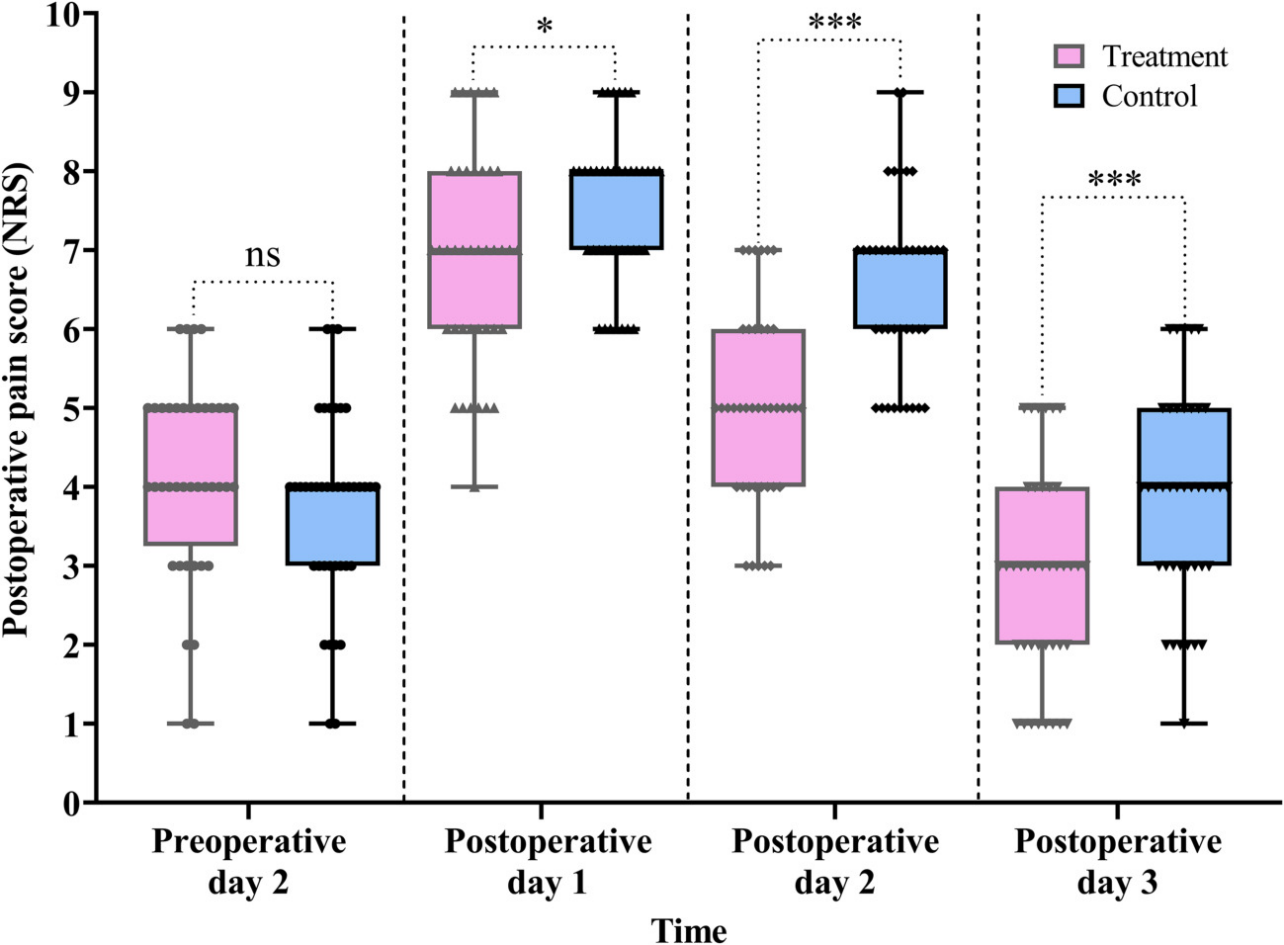
Comparison of the pre- and postoperative pain scores between the treatment and control groups. Data are expressed as the median (IQR) (*n* = 40/group). Statistical significance was defined as ns, not statistically, *P < 0.05, and ***P < 0.001.

As shown in Table 2 and Figure 5, the treatment group demonstrated significantly lower NRS scores compared with the control group on POD 1 (difference, −1.0, 95% CI: −1.0 to 0.0, *P* *=* 0.031), POD 2 (difference, −1.0, 95% CI: −2.0 to −1.0, *P* < 0.001), and POD 3 (difference, −1.0, 95% CI: −2.0 to 0.0, *P* *=* 0.001).

Furthermore, the Scheirer–Ray–Hare test revealed a significant main effect of group (*H* = 15.855, *P* < 0.001) and time (*H* = 142.752, *P* < 0.001) on pain score reduction. However, the interaction between group and time was not statistically significant (*H* = 2.820, *P* *=* 0.244) (for detailed results, see Table S3 in the Supplementary Material).

#### 3.3.3 Postoperative nausea and vomiting score

Postoperative nausea and vomiting are commonly triggered by the surgical trauma, which can induce systemic dysregulation of neuromodulatory networks. In particular, sympathovagal imbalance disrupts neurohumoral control of GI function and gastroduodenal coordination.

**Figure 6:**
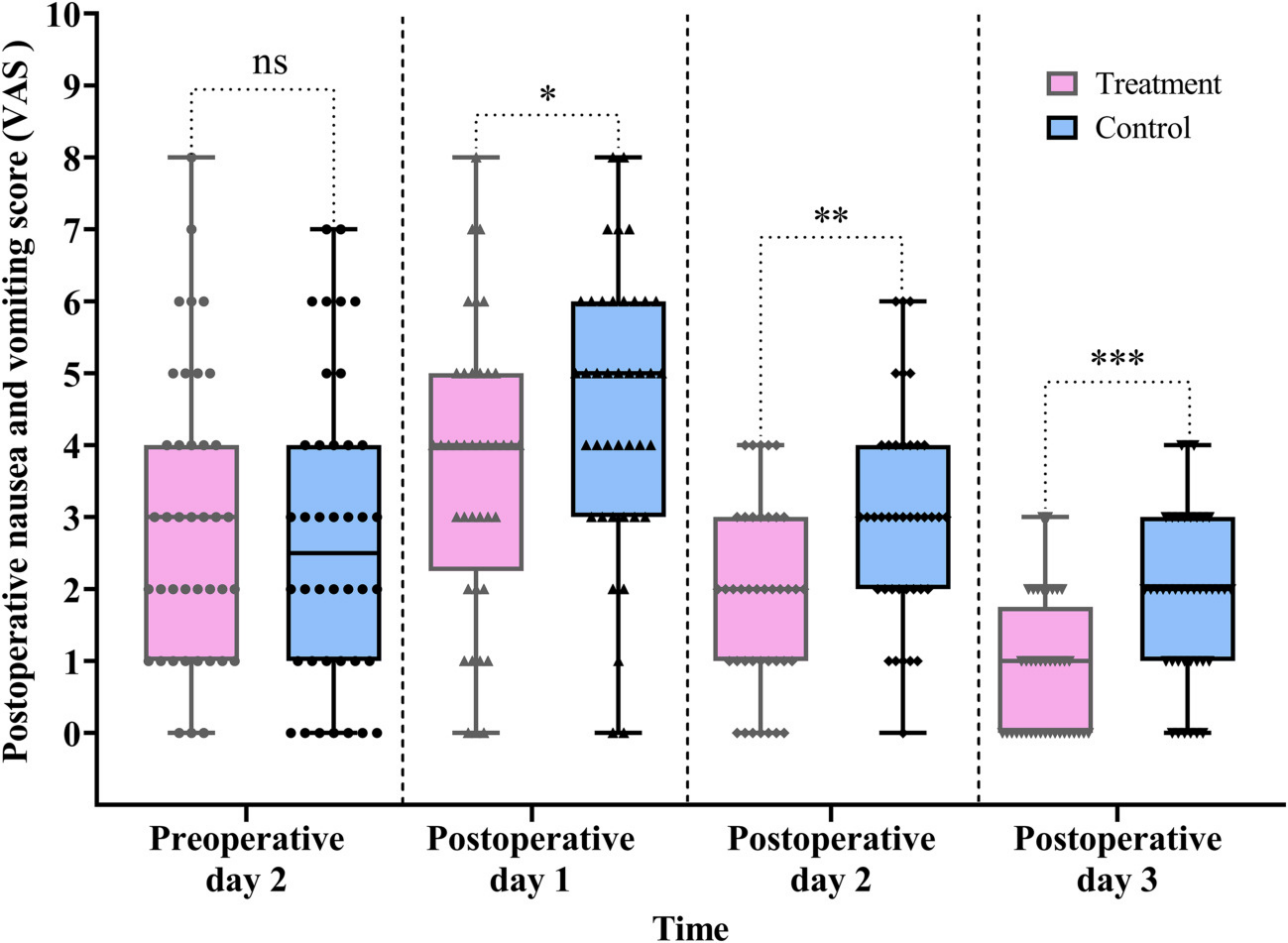
Comparison of the pre- and postoperative nausea and vomiting scores between the treatment and control groups. Data are expressed as the median (IQR) (*n* = 40/group). Statistical significance was defined as ns, not statistically, *P < 0.05, **P < 0.01, and ***P < 0.001.

In this study, nausea and vomiting were assessed using the VAS. As shown in Table 2 and Figure 6, VAS scores in the treatment group were significantly lower than those in the control group on all three postoperative days: POD 1 (difference, −1.0, 95% CI: −2.0 to 0.0, *P* *=* 0.034), POD 2 (difference, −1.0, 95% CI: −2.0 to −1.0, *P* *=* 0.001), and POD 3 (difference, −1.0, 95% CI: −2.0 to −1.0, *P* < 0.001).

The Scheirer–Ray–Hare test revealed a significant main effect of group (*H* = 18.157, *P* < 0.001) and time (*H* = 79.931, *P* < 0.001) on VAS scores. However, the interaction between group and time was not statistically significant (*H* = 0.918, *P* *=* 0.632) (for detailed results, see Table S4 in the Supplementary Material).

#### 3.3.4 Postoperative abdominal distention score

Evaluating postoperative abdominal distension is important for assessing early GI function recovery. In this study, abdominal distension was measured using the GSRS.

**Figure 7:**
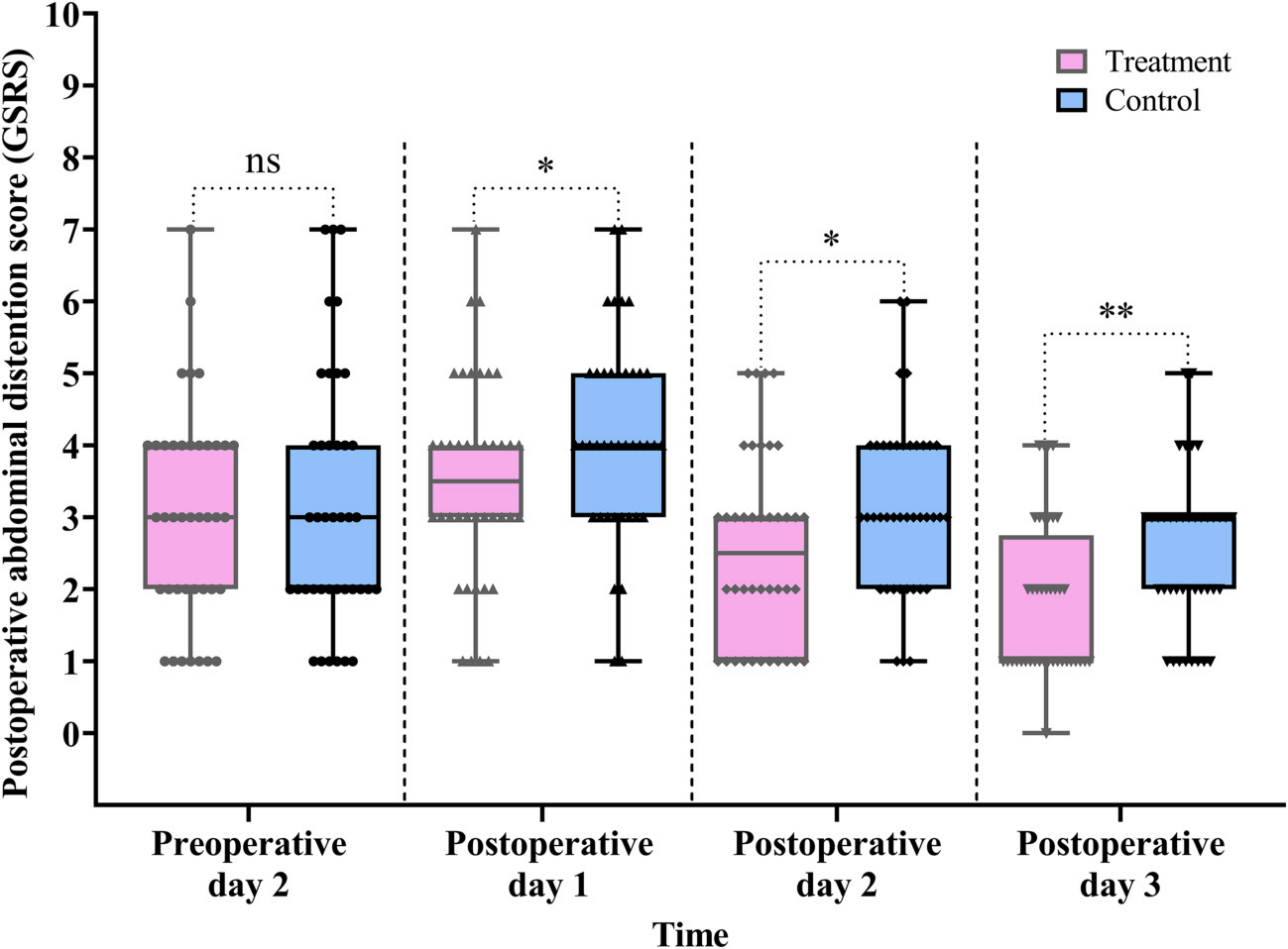
Comparison of the pre- and postoperative abdominal distention scores between the treatment and control groups. Data are expressed as the median (IQR) (*n* = 40/group). Statistical significance was defined as ns, not statistically, *P < 0.05, and **P < 0.01.

As shown in Table 2 and Figure 7, GSRS scores were significantly lower in the treatment group compared with the control group on POD 1, 2, and 3: POD 1 (difference, −1.0, 95% CI: −1.0 to 0.0, *P* *=* 0.047), POD 2 (difference, −1.0, 95% CI: −1.0 to 0.0, *P* *=* 0.028), and POD 3 (difference, −1.0, 95% CI: −1.0 to 0.0, *P* *=* 0.003) (Table 2, Figure 7).

The Scheirer–Ray–Hare test showed a significant main effect of group (*H* = 12.472, *P* < 0.001) and time (*H* = 51.087, *P* < 0.001) on GSRS scores. However, the interaction between group and time was not statistically significant (*H* = 0.143, *P* *=* 0.931) (for detailed results, see Table S5 in the Supplementary Material).

#### 3.3.5 Postoperative hospital stay

**Figure 8:**
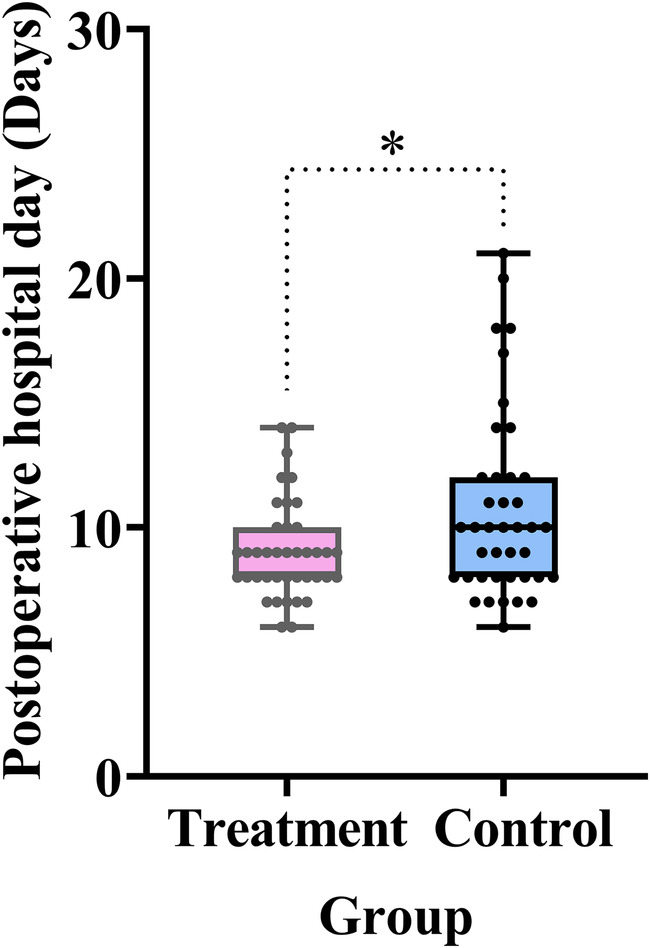
Comparison of the postoperative hospital stay between the treatment and control groups. Data are expressed as the mean (SD) (*n* = 40/group). Statistical significance was defined as *P < 0.05.

A shortened postoperative hospital stay directly reflects enhanced recovery of GI function. As shown in Table 2 and Figure 8, the treatment group had a significantly shorter postoperative hospital stay compared with the control group (difference, −1.7 days, 95% CI: −3.0 to 0.3, *P* *=* 0.015).

#### 3.3.6 Postoperative complications

Among the 80 participants included in the study, six cases of postoperative complications were reported. In the treatment group, two patients (5.0%) experienced complications (both were pulmonary infections), while in the control group, four patients (10.0%) developed complications (all pulmonary infections). There was no statistically significant difference in the incidence of postoperative complications between the two groups (difference, −5.0%, 95% CI: −18.6 to 8.0, *P* *=* 0.396) (Table 2).

#### 3.3.7 Safety evaluation

No treatment-related adverse events were observed in the treatment group, indicating that thumbtack needle therapy was safe and well-tolerated.

#### 3.3.8 Overall response rate

**Figure 9:**
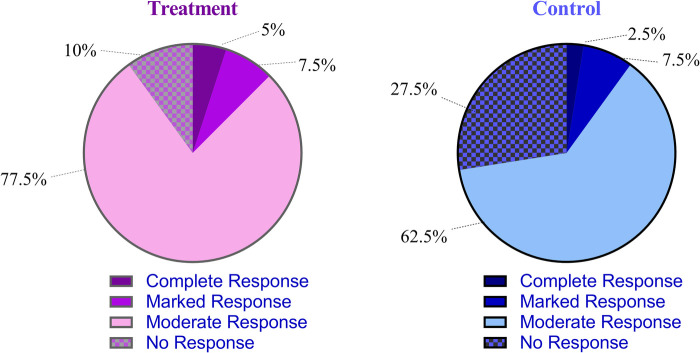
Comparison of the overall response rate between the treatment and control groups. The overall response rate was defined as the sum of complete, marked, and moderate responses according to the Rome IV criteria.

The overall response rate in the treatment group was significantly higher than that in the control group (difference, 17.5%, 95% CI: 0.2 to 34.0, *P* *=* 0.046) (Table 2, Figure 9).

## 4 Discussion

PGD remains a common and challenging complication after gastric cancer surgery. Currently, modern medicine lacks specific treatments for PGD, with management largely relying on prokinetic agents. However, prolonged or high-dose use of these medications may lead to adverse effects such as extrapyramidal symptoms, fatigue, drowsiness, diarrhea, abdominal pain, dry mouth, rash, and dizziness. Although ERAS protocols have improved overall perioperative recovery, they remain insufficient in shortening the duration of early postoperative ileus (21, 24). Thus, exploring novel and effective strategies to accelerate early GI function recovery is of pressing clinical importance.

In recent years, the integration of acupuncture into ERAS pathways has gained momentum, particularly in GI surgeries. Acupuncture has been reported to preserve intestinal mucosal barrier integrity, alleviate postoperative pain, and reduce the need for anesthetics. According to modern medical theories, PGD is triggered by surgical trauma, initiating a cascade of physiological disruptions: localized ischemia–reperfusion injury, impaired microcirculatory perfusion, systemic inflammation, and mitochondrial dysfunction. These changes undermine cellular energy metabolism, while sympathetic overactivity suppresses vagal tone, leading to sphincter dyssynergia and motility failure. This dysfunction promotes small intestinal bacterial overgrowth and mucosal barrier breakdown, facilitating endotoxin translocation and perpetuating a cycle of GI paralysis and systemic inflammation (25).

Acupuncture exerts therapeutic effects by enhancing vagal tone and mitigating neurogenic inflammation, thus addressing both the neural and immune dysregulation underlying PGD. This aligns with traditional Chinese medicine principles, where the unimpeded flow of Qi is considered foundational to functional recovery (23).

In this study, we selected five classical acupoints: Neiguan (PC6), Zusanli (ST36), Shangjuxu (ST37), Hegu (LI4), and Sanyinjiao (SP6) (22–26):

• Neiguan is a key point for regulating the triple energizer (San Jiao) and is widely used to treat postoperative nausea and vomiting.
• Zusanli, the lower He-sea point of the stomach, harmonizes the spleen and stomach, regulates Qi, and is a primary point for GI disorders.
• Shangjuxu, the lower He-sea point of the large intestine, promotes intestinal peristalsis and relieves food retention.
• Hegu, regulates stomach Qi and alleviates retching and epigastric discomfort.
• Sanyinjiao, belonging to the spleen meridian, nourishes Yin, harmonizes the stomach, and improves intestinal motility.

The synergistic stimulation of these acupoints facilitates GI neuromodulation, enhances enteric function, and contributes to faster postoperative recovery.

The continuous and stable subcutaneous stimulation provided by thumbtack needles enables sustained activation of meridians, ultimately contributing to the restoration of visceral functional homeostasis. Compared with traditional filiform needle acupuncture, thumbtack needle embedding offers several advantages: it is convenient, safe, is minimally invasive, and allows patients to remain mobile. Patients can apply gentle pressure to the embedded needles on their own, independent of fixed treatment schedules. This approach compensates for the short duration of conventional acupuncture sessions while avoiding the discomfort often associated with filiform needle insertion (26).

In acupuncture clinical trials, two main types of control interventions are commonly employed: non-insertion and needle-insertion sham controls. These are designed to maintain patient blinding and minimize non-specific effects associated with psychological or placebo responses, thereby allowing for a more accurate evaluation of the specific therapeutic effects attributable to needle insertion. Non-insertion sham controls aim to completely eliminate the physiological effects of acupuncture; however, they are difficult to implement effectively. Because the skin is not penetrated, patients can often distinguish the intervention, compromising blinding. In addition, even light skin contact can induce sensory stimulation and physiological responses, potentially confounding the results. Consequently, all forms of sham acupuncture may inadvertently elicit somatosensory and biological effects, which can influence study outcomes and reduce the contrast between groups (27–29). In this study, we opted for a blank control (i.e., no acupuncture intervention) to evaluate the overall clinical efficacy of thumbtack needles in a real-world setting.

This study demonstrated that the treatment group experienced significantly earlier recovery in several key indicators of GI function, including time to bowel sound recovery, first flatus, first defecation, and removal of both nasogastric tube and intra-abdominal drains, compared with the control group. These findings suggest that thumbtack needle therapy applied to the selected acupoints can effectively facilitate early GI functional recovery following laparoscopic gastrectomy for gastric cancer. In addition, the evaluation of postoperative GI symptoms provided valuable clinical insight. Using symptom scores recorded on POD 1–3, we compared abdominal pain, distention, nausea, and vomiting between the two groups. The treatment group consistently showed lower symptom scores across all time points, indicating that thumbtack needle therapy not only accelerates physiological recovery but also significantly alleviates common postoperative GI symptoms associated with laparoscopic gastric cancer surgery.

We also compared the incidence of postoperative complications between the two groups. The results revealed no significant differences in the occurrence of complications such as postoperative bleeding, infection, and anastomotic leakage. These findings suggest that thumbtack needle therapy does not increase the risk of postoperative complications. Furthermore, no adverse reactions such as syncope, needle retention, or needle breakage were reported among participants. No serious adverse events occurred throughout the study, indicating that thumbtack needle therapy is a safe intervention in the postoperative setting.

Moreover, clinical efficacy was assessed using the internationally recognized Rome IV criteria (2016). The results showed that the overall response rate was significantly higher in the treatment group than that in the control group. In addition, the treatment group had a significantly shorter postoperative hospital stay compared with the control group.

Our study also has several limitations. First, it lacked objective and quantitative outcome measures, and no pre- and postoperative biochemical indicators were collected for comparison. Second, the mechanisms by which the thumbtack needle promotes early GI function recovery after laparoscopic gastric cancer surgery were not explored. Third, the study included only 80 participants and was conducted at a single center, which may introduce potential biases and limit the generalizability of the findings. However, compared with multicenter studies, single-center trials allow for better quality control and ensure greater internal validity. These limitations should be addressed in future multicenter, larger-scale studies incorporating objective biomarkers and mechanistic investigations.

## 5 Conclusion

The results of this study indicate that thumbtack needle therapy applied to bilateral Neiguan (PC6), Zusanli (ST36), Shangjuxu (ST37), Hegu (LI4), and Sanyinjiao (SP6) is a safe and effective intervention for promoting early GI recovery following laparoscopic radical gastrectomy. This approach significantly shortens the time to postoperative bowel sound recovery, first flatus, first defecation, and the removal of nasogastric tube and intra-abdominal drains. In addition, it demonstrates superior efficacy over conventional care in alleviating postoperative symptoms such as nausea and vomiting, abdominal pain, and distention. The therapy also contributes to reduced hospital stay and yields a significantly higher overall response rate, supporting its clinical value as a complementary treatment within ERAS protocols.

## Data Availability

The datasets presented in this study can be found in online repositories. The names of the repository/repositories and accession number(s) can be found in the article/Supplementary Material.
