# Supplementary material for: Effect of the thumbtack needle on gastrointestinal function recovery after laparoscopic radical gastrectomy for gastric cancer with the concept of enhanced recovery after surgery: a randomized controlled trial
Source: Front Surg. 2025 Sep 18;12:1612766. doi: 10.3389/fsurg.2025.1612766 (PMC12488703; doi:10.3389/fsurg.2025.1612766)
Supplement: Supplementary file 1 [file Supplementaryfile1.docx]

**Supplementary Online Content**

**Figure S1** Acupoint Locations

**Table S1** Acupoint Locations

**Table S2** Assessment criteria of Primary outcomes and Second outcomes

**Table S3** Scheirer Ray Hare Test of Postoperative Pain Score

**Table S4** Scheirer Ray Hare Test of Postoperative nausea and vomiting score

**Table S5** Scheirer Ray Hare Test of Postoperative abdominal distention score

This supplementary material has been provided by the authors to give readers additional information about their work

**Figure S1** Acupoint Locations

**
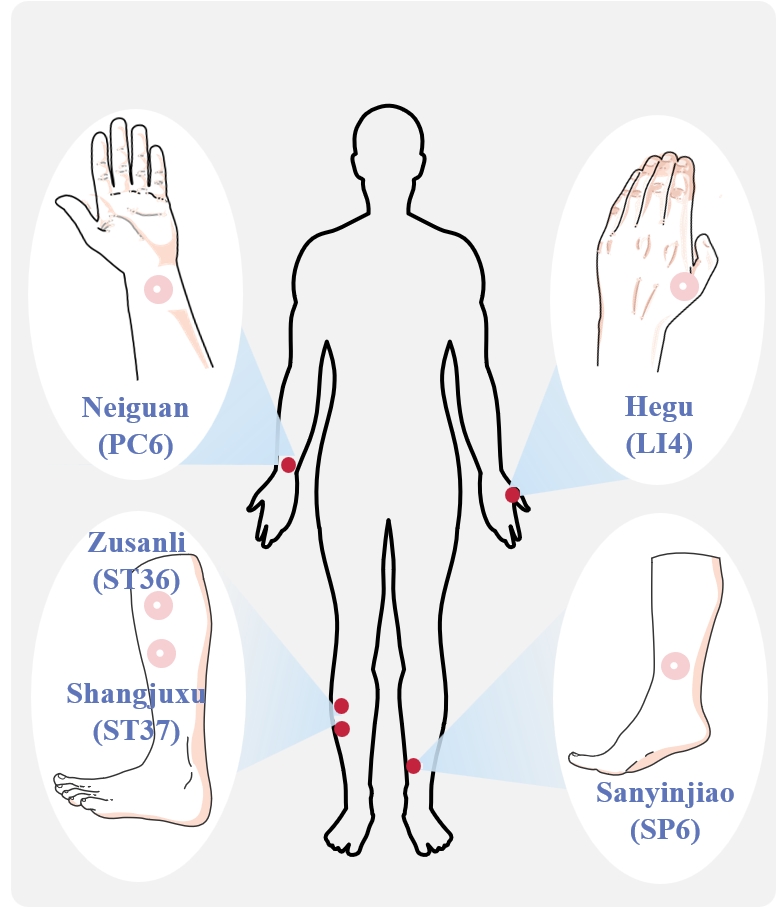
**

**Table S1** Acupoint Locations

| **Acupoint** | **Locations** |
| --- | --- |
| **Neiguan (PC6)** | on the palm side of the forearm, 2 cun above the transverse crease of the wrist, between the tendons of the palmar longus and the radial flexor muscle of the wrist. |
| **Zusanli (ST36)** | on the outer side of the lower leg, on the line connecting Dubi (ST35) and Jiexi (ST41), 3 cun below Dubi |
| **Shangjuxu (ST37)** | on the outer side of the lower leg, on the line connecting Dubi (ST35) and Jiexi (ST41), 6 cun below Dubi |
| **Hegu (LI4)** | on the back of the hand, between the first and second metacarpal bones, at the midpoint of the radial side of the second metacarpal bone. |
| **Sanyinjiao (SP6)** | on the inner side of the lower leg, 3 cun above the tip of the medial malleolus, behind the medial edge of the tibia |

**Table S2** Assessment criteria of Primary outcomes and Second outcomes

| **Outcomes** | | | **Assessment criteria** |
| --- | --- | --- | --- |
| **Primary outcomes** | Time to bowel sound recovery | | The time from the end of the surgery to the restoration of bowel sounds to their preoperative normal state. Detect method: assessed via auscultation by trained medical staff every 4-6 hours after surgery. Auscultation was performed in all four abdominal quadrants, and recovery was defined as the presence of ≥4 bowel sounds per minute in at least two quadrants. The recovery time was recorded based on the first assessment that met these criteria. |
|  | Time to first flatus | | The time from the end of the surgery to the first flatus. |
| **Second outcomes** | Time to first defecation | | The time from the end of the surgery to the first defecation. |
|  | Time to nasogastric tube removal | | The time from the end of the surgery until the determination that the nasogastric tube can be removed. Removal criteria: performed when patients had no active vomiting or abdominal distension, gastric residual volume was <200 ml in 24 hours, bowel sounds had returned, and there were no signs of vomiting, abdominal distension, or suspected obstruction. |
|  | Time to intra-abdominal drains removal | | The time from the end of the surgery until the determination that the intra-abdominal drains can be removed. Removal criteria: performed when the drain output was <50 ml/day of non-bloody, non-bilious fluid, without signs of infection or anastomotic leakage. |
|  | Postoperative pain score | | NRS was employed to assess the pain status of two groups. The NRS categorizes pain into 11 levels: 0 points: no pain; 1-3 points: mild pain; 4-6 points: moderate pain; 7-10 points: severe pain. |
|  | Postoperative nausea and vomiting score | | VAS was used to assess nausea and vomiting conditions of two groups, utilizing a movable ruler approximately 10 cm long, marked with 10 graduations, with 0 at one end and 10 at the other end. A score of 0 indicates no nausea or vomiting, while 10 represents the most severe nausea or vomiting. |
|  | Postoperative abdominal distention score | | GSRS was utilized to evaluate the abdominal distention condition of two groups. GSRS categorizes abdominal distention into 7 levels, with 1 point denoting no discomfort, 2-3 points indicating mild abdominal distention, 4-5 points representing moderate abdominal distention, and 6-7 points indicating severe abdominal distention. |
|  | Postoperative hospital stay | | Duration from the end of the surgery until the patient's discharge, measured in days. Discharge criteria were standardized across both groups and included: (1) Return of bowel function, no positive findings on abdominal examination; (2) Return to normal dietary; (3) Normal body temperature was observed; (4) No signs of complications (e.g., bleeding, infection, anastomotic leakage); (5) Normal or recovering lab parameters (e.g., WBC, CRP). |
|  | Postoperative complications | | Early complications occurring during the hospitalization period after subtotal or partial gastrectomy in gastric cancer, including infection, bleeding, delayed gastric emptying, anastomotic leakage, etc. |
|  | Safety evaluation | | Including dizziness from needling, retained or broken needles, intolerable needle pain (NRS≥7), localized hematoma, and other discomforts post-acupuncture treatment (referring to sustained symptoms such as nausea, vomiting, dizziness, pain, palpitations, and loss of appetite persisting for at least 1 hour after acupuncture treatment); and unforeseen adverse events. |
|  | Overall response rate | Complete response | Normal restoration of flatus and defecation within 24 hours postoperatively, complete bowel sounds heard upon auscultation at 4-5 times/minute, no abdominal distension, only slight pain occurring during coughing, no nausea, no vomiting. |
|  |  | Marked response | Flatus or defecation within 24-48 hours postoperatively, weaker bowel sounds at 2-3 times/minute, pain occurring during deep breathing, slight nausea without vomiting. |
|  |  | Moderate response | Flatus or defecation within 48-72 hours postoperatively, abnormal bowel sounds at 1-2 times/minute, tolerable abdominal distension and pain at rest, significant nausea without emesis. |
|  |  | No response | No flatus or defecation beyond 72 hours postoperatively, disappearance or extreme weakness of bowel sounds, no significant improvement or worsening of symptoms before and after treatment, obvious abdominal distension and pain, accompanied by nausea and vomiting. |

**Table S3** Scheirer Ray Hare Test of Postoperative Pain Score

|  | Df | Sum Sq | H | P. value |
| --- | --- | --- | --- | --- |
| Time | 2 | 674489 | 142.752 | ＜0.001 |
| Group | 1 | 74871 | 15.8546 | ＜0.001 |
| Time: Group | 2 | 13326 | 2.820 | 0.244 |
| Residuals | 234 | 366563 |  |  |

**Table S4** Scheirer Ray Hare Test of Postoperative nausea and vomiting score

|  | Df | Sum Sq | H | P. value |
| --- | --- | --- | --- | --- |
| Time | 2 | 375738 | 79.931 | ＜0.001 |
| Group | 1 | 85353 | 18.157 | ＜0.001 |
| Time: Group | 2 | 4313 | 0.918 | 0.632 |
| Residuals | 234 | 658082 |  |  |

**Table S5** Scheirer Ray Hare Test of Postoperative abdominal distention score

|  | Df | Sum Sq | H | P. value |
| --- | --- | --- | --- | --- |
| Time | 2 | 235669 | 51.087 | ＜0.001 |
| Group | 1 | 57536 | 12.472 | ＜0.001 |
| Time: Group | 2 | 660 | 0.143 | 0.931 |
| Residuals | 234 | 808660 |  |  |
