# Supplementary material for: Effect of the thumbtack needle on gastrointestinal function recovery after laparoscopic radical gastrectomy for gastric cancer with the concept of enhanced recovery after surgery: a randomized controlled trial
Source: Front Surg. 2025 Sep 18;12:1612766. doi: 10.3389/fsurg.2025.1612766 (PMC12488703; doi:10.3389/fsurg.2025.1612766)
Supplement: Supplementary file 3 [file Supplementaryfile3.docx]

**Effect of the thumb-tack needle on gastrointestinal function recovery after laparoscopic radical gastrectomy for gastric cancer with the concept of enhanced recovery after surgery: A randomized controlled trial**

The Affiliated Hospital of Guizhou Medical University

**Data:**

Original protocol date: Jun 10, 2023

Amendment date: Mar 22, 2024

**Clinical sites:**

The Affiliated Hospital of Guizhou Medical University

**Data Management and Statistical Centers:**

The Affiliated Hospital of Guizhou Medical University

**Integrity statement**

This study ensures that all procedures are strictly conducted according to the trial protocol and the data recorded is authentic. There are no conflicts of interest in this study.

**Contents**

[**1.** **Study Contact and Organization** 1](#_Toc177314621)

[**1.1** **Study Contacts** 1](#_Toc177314622)

[**1.2** **Investigators** 1](#_Toc177314623)

[**2.** **Study Design** 1](#_Toc177314624)

[**2.1** **Background** 1](#_Toc177314625)

[**2.2** **Study Objective** 3](#_Toc177314626)

[**2.3** **Methodology** 3](#_Toc177314627)

[**3.** **Interventions** 5](#_Toc177314628)

[**3.1** **ERAS perioperative management** 5](#_Toc177314629)

[**3.2** **Treatment group** 5](#_Toc177314630)

[**3.3** **Control group** 6](#_Toc177314631)

[**4.** **Outcome measurement** 6](#_Toc177314632)

[**4.1** **Primary outcomes** 6](#_Toc177314633)

[**4.2** **Secondary outcomes** 7](#_Toc177314634)

[**5.** **Ethical Principle** 8](#_Toc177314635)

[**6.** **Statistical Analysis** 8](#_Toc177314636)

[**7.** **Revision history** 8](#_Toc177314637)

[**8.** **Funding** 9](#_Toc177314638)

[**References** 9](#_Toc177314639)

1. **Study Contact and Organization**
   1. **Study Contacts**

Principal Investigator for study,

Professor Qian Wang.

The Affiliated Hospital of Guizhou Medical University, No.28, Guiyi St, Yunyan District, Guiyang 550004, Guizhou, China.

Email: [wq5969@sina.com](mailto:wq5969@sina.com)

- 1. **Investigators**

Qian Wang, Professor, supervisor of experiment

Email: [wq5969@sina.com](mailto:wq5969@sina.com)

Shuai Guo, MD, Concept and design of experiment

Email: [guoshuai@gmc.edu.cn](mailto:guoshuai@gmc.edu.cn)

Xiang-Ping Lin, PhD, Concept and design of experiment, Implementation of experiment

Email: [xiangping902@163.com](mailto:xiangping902@163.com)

Xiang-Ren Jin, PhD, Implementation of experiment, Administrative, technical, or material support

Email: [411084152@qq.com](mailto:411084152@qq.com)

Pei Li, MD, Implementation of experiment, Administrative, technical, or material support

Email: [18984145332@163.com](mailto:18984145332@163.com)

Kang-Xiu Tuo, MD, Outcome evaluation, Collection, analysis, or interpretation of data Email: [294682226@qq.com](mailto:294682226@qq.com)

Wei-Wei Yang, MD, Outcome evaluation, Collection, analysis, or interpretation of data Email: [609153003@qq.com](mailto:609153003@qq.com)

1. **Study Design**
   1. **Background**

Gastric cancer (GC) refers to a malignant tumor of the gastric mucosal epithelial cells and is one of the most common cancers worldwide, ranking fifth in incidence and fourth in mortality^1,2^. The 5-year survival rate for patients with gastric cancer after surgery is only 30%-40%^3^. In China, the incidence of gastric cancer ranks second, following lung cancer, and it ranks third in mortality. Due to factors such as living environment, dietary habits, and the gradual popularization of gastroscopy, the number of gastric cancer patients in China has increased in recent years, with a trend towards younger age. The principle of gastric cancer treatment is comprehensive therapy, primarily surgical, supplemented by multidisciplinary approaches^4-5^. Surgery remains the main treatment method for gastric cancer. However, this severely impacts the postoperative quality of life for almost all gastric cancer patients. The main postoperative complication of gastric cancer is gastrointestinal (GI) dysfunction, which not only prolongs the time for postoperative defecation and eating but also easily leads to postoperative malnutrition, severely affecting the recovery and even survival time of gastric cancer patients. Therefore, it is crucial to restore GI function as soon as possible, alleviate postoperative symptoms, and improve the quality of life for postoperative gastric cancer patients.

In recent years, the concept of Enhanced Recovery After Surgery (ERAS) has gradually gained promotion and popularity in the field of surgery. However, various management measures are still in the exploratory stage, and the application of ERAS during the perioperative period of gastric cancer radical surgery requires further exploration^6-7^. Integrating traditional Chinese medicine with ERAS represents a new idea and direction for the development of ERAS, combining the concepts of both traditional Chinese medicine and ERAS. Acupuncture has unique advantages in promoting the recovery of GI function in patients after surgery^8-11^. It can not only enhance GI function recovery but also reduce preoperative anxiety, intraoperative stress, and postoperative complications, which are difficult to achieve with the existing ERAS management measures alone. With the ERAS concept, acupuncture's role in perioperative management aligns with the holistic treatment principles of traditional Chinese medicine, which views disease and the body as an integrated whole rather than focusing on a specific part or symptom. This has great importance for postoperative recovery.

Currently, the modern medical treatment of postoperative GI dysfunction mainly includes the use of prokinetic drugs, GI decompression, and nutritional support^12^. However, these methods often result in poor clinical outcomes, significant side effects, and high costs. While current enhanced recovery techniques during the perioperative period have accelerated the overall recovery of patients, they have not effectively reduced the time required for early postoperative GI function recovery. After radical gastrectomy, there is still a lack of effective interventions for early GI dysfunction. The urgent issue in clinical practice is to explore new and effective methods to promote early GI function recovery after radical gastrectomy. In recent years, some clinical studies have found that acupuncture can regulate GI motility, stabilize gut microbiota, and enhance the recovery of GI function^13^.

We hypothesized that thumb-tack needle treatment would promote early recovery of GI function following laparoscopic radical gastrectomy. Therefore, we design this study to investigate the efficacy and safety of thumb-tack needle treatment in patients undergoing laparoscopic gastrectomy for gastric cancer.

- 1. **Study Objective**

To investigate whether thumb-tack needle treatment can promote early recovery of GI function following laparoscopic radical gastrectomy and observe its safety.

- 1. **Methodology**
     1. **Trial Design**

This prospective, single-center, randomized controlled, and non-blinded trial was performed at the gastroenterology department in the Affiliated Hospital of Guizhou Medical University. This trial has been approved by the Ethics Committee of the Affiliated Hospital of Guizhou Medical University (Approval No. 2023[6609]). All eligible participants provided written informed consent before enrolling in the trial. This trial was registered with Chinese Clinical Trial Registry (ChiCTR2400084712). This study followed the Consolidated Standards of Reporting Trials (CONSORT) reporting guideline.

- - 1. **Patients**
       1. Patients Recruitment, Screening

The participants will be recruited from the gastric cancer undergoing laparoscopic surgery of the Affiliated Hospital of Guizhou Medical University. Participants were randomly assigned at random (1:1) to either the Treatment group or the Control group. An independent researcher will conduct face-to-face interviews with the participants to explain the study, and those who volunteer to participate will be required to sign consent forms. After a baseline screening visit, participants who meet the inclusion criteria will be able to participate in the study. The participant flow was shown in Figure 1.

- - - 1. Inclusion Criteria

(1) Age 18-70 years, regardless of gender;

(2) Histologically confirmed gastric cancer based on gastroscopy and pathological examination;

(3) Scheduled for laparoscopic radical gastrectomy;

(4) Voluntary participation with signed informed consent;

(5) Acceptance of acupuncture therapy.

- - - 1. Exclusion Criteria

(1) Known metal allergy or significant needle phobia;

(2) Surgical incisions or scarring in the meridian areas corresponding to Neiguan (PC6), Zusanli (ST36), Shangjuxu (ST37), Hegu (LI4), or Sanyinjiao (SP6);

(3) Local skin infections at the aforementioned acupoints;

(4) Inability to comprehend or complete the Visual Analogue Scale (VAS), Numeric Rating Scale (NRS), or Gastrointestinal Symptom Rating Scale (GSRS);

(5) Conversion to open surgery;

(6) Occurrence of serious adverse events (AEs);

(7) Judged by investigators to be unsuitable for participation.

- - 1. **Randomization and Blinding**

Eligible patients were randomly assigned to either the treatment group or control group in a 1:1 ratio using SPSS version 26.0 to generate the randomization sequence. The allocation results were placed sequentially into sealed, opaque envelopes, which were prepared and managed by the individual who generated the randomization sequence. An envelope was opened only after a participant met all inclusion and exclusion criteria and provided informed consent. Perioperative management was then conducted according to the group assignment indicated in the envelope.

The researcher who generated the randomization sequence was not involved in participant enrollment or group assignment. Furthermore, the individual responsible for generating and maintaining the randomization list was not involved in any other aspect of the study.

- - 1. **Sample Size**

In this study, the expected difference between the treatment and control groups was estimated based on a previous study^14^, which reported a postoperative time to first flatus of 32.03 ± 8.42 hours in the acupuncture group versus 38.03 ± 7.31 hours in the control group. This yielded an expected mean difference of approximately 6 hours, which was used as the basis for sample size calculation. Assuming a two-sided significance level of α = 0.05 and a power (1–β) of 80%, the corresponding Z values for a bilateral test were Z_α/2_ = 1.96 and Z_β_ = 0.84. The ratio between the treatment group and control group was set at 1:1. The sample size was calculated using the following formula:$n={(Z_{\alpha/2}+Z_{\beta})}^{2}\cdot{(\sigma_{1}^{2}+\sigma_{2}^{2})}^{2}/\delta^{2}$. A sample size of 36 per group was calculated, and with an estimated 10% dropout rate, 40 patients were enrolled in each group (total N = 80).


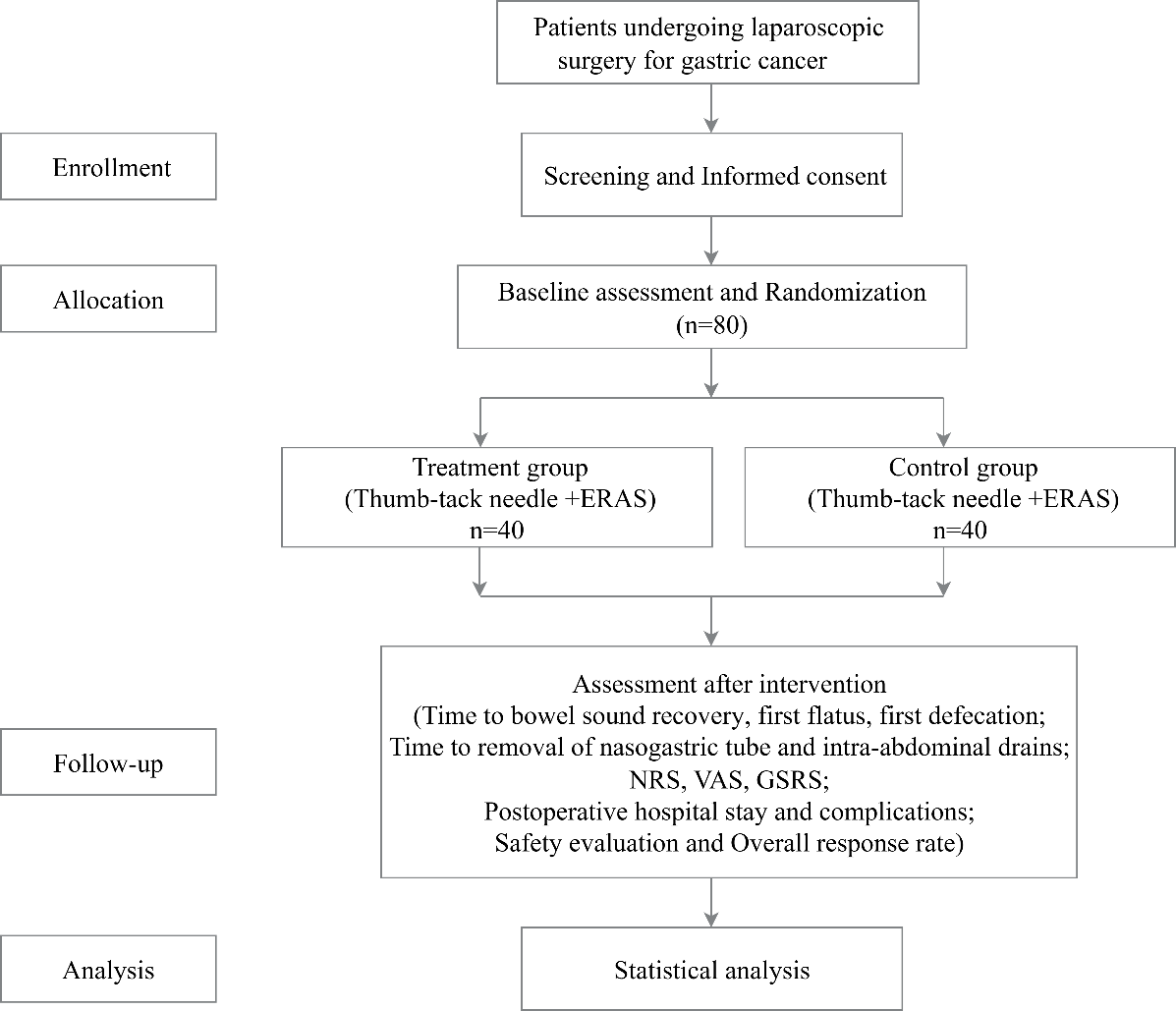


Figure 1 Flow chart

1. **Interventions**
   1. **ERAS perioperative management**
      1. **Preoperative period**
         1. Preoperative education: On the day of admission, patients were educated on the components and anticipated benefits of ERAS-based perioperative management to ensure their understanding and cooperation, as well as that of their families.
         2. Nutritional risk assessment: For patients with nutritional risk (NRS2002 ≥ 3 points), enteral or parenteral nutrition is provided based on whether the patient is fasting.
         3. Bowel preparation: Mechanical bowel preparation was not performed.
         4. Fasting: Patients were instructed to fast from solid food for 6 hours and from clear liquids for 2 hours before surgery.
         5. Perioperative prophylactic antithrombotic therapy: The Caprini Thrombosis Risk Assessment Scale was used to evaluate the risk of venous thromboembolism at admission. Patients with a Caprini score of 0 required no intervention. Those with a score of 1-2 received mechanical prophylaxis using intermittent pneumatic compression devices. Patients with a score of 3-4 and no high bleeding risk were given low molecular weight heparin (LMWH) for prophylaxis, for at least 7 days. Patients with a score ≥ 5 and no high bleeding risk received combined therapy with both mechanical compression and LMWH. Postoperatively, patients with a Caprini score of 1–4 were treated with both pneumatic compression and LMWH for more than 7 days. For patients with a Caprini score ≥ 5, LMWH prophylaxis was continued for 4 weeks after surgery.
         6. Prophylactic antibiotic use: Antibiotics were administered 30 minutes before surgery.
      2. **Intraoperative period**
         1. Anesthesia management: General and intravenous combined anesthesia.
         2. Temperature management: Intraoperative body temperature was monitored. Warm distilled water was used for peritoneal lavage, and a warming blower was applied to maintain normothermia during surgery.
         3. Fluid management: No fluid restriction.
         4. Intra-abdominal drains management: Selectively placed based on the patient’s intraoperative surgical condition, and removed early, within 1-2 postoperative days.
         5. Nasogastric tube management: Inserted intraoperatively, and removed within 24 hours postoperatively if the anastomosis was deemed satisfactory.
         6. Urinary catheter management: Inserted intraoperatively, and removed early, within 1-2 postoperative days.
      3. **Postoperative period**
         1. Pain management: Adequate postoperative analgesia was provided using a multimodal analgesic strategy, including low-dose opioids combined with NSAIDs, along with patient-controlled intravenous analgesia.
         2. Prevention and treatment of nausea and vomiting: Antiemetic drugs were administered intravenously.
         3. Dietary guidance: Except in patients with impaired intestinal function, anastomotic leakage, bowel obstruction, or high risk of gastroparesis, we initiate orally ingested nutritional support within 24 hours after surgery
         4. Mobilization out of bed 24 hours after surgery.
         5. Fluid management: A goal-directed fluid therapy approach, which aims to maintain appropriate tissue perfusion and organ function while avoiding both hypovolemia-related complications and volume overload.
   2. **Treatment group**
      1. **Equipment:** Disposable, sterile, thumb-tack needles (Φ0.20 × 1.0mm, Hwato, Suzhou Medical Appliance Factory, China)
      2. **Acupoints selection:** Building upon the standard ERAS perioperative management (3.1), patients in the treatment group will be treated bilaterally at five acupoints. (shown in figure2) (Referring to the 2021 National Standard - GB/T 12346-2021 "Nomenclature and location of meridian points").
         1. Neiguan (PC6): It is located on the palm side of the forearm, on the line between Quze (PC3) and Dalingshen (PC7), 2 cun above the transverse crease of the wrist, between the tendons of the palmar longus and the radial flexor muscle of the wrist.
         2. Zusanli (ST36): It is located on the outer side of the lower leg, on the line connecting Dubi (ST35) and Jiexi (ST41), 3 cun below Dubi.
         3. Shangjuxu (ST37): It is located on the outer side of the lower leg, on the line connecting Dubi (ST35) and Jiexi (ST41), 6 cun below Dubi.
         4. Hegu (LI4): It is located on the back of the hand, between the first and second metacarpal bones, at the midpoint of the radial side of the second metacarpal bone.
         5. Sanyinjiao (SP6): It is located on the inner side of the lower leg, 3 cun above the tip of the medial malleolus, behind the medial edge of the tibia.
      3. **Procedure:** Before application, the skin at the acupoint sites was disinfected using 75% alcohol. A disposable thumb-tack needle was then applied to each acupoint with vertical pressure using the thumb or index finger, starting gently and increasing until a tingling sensation (deqi) was achieved. Each session involved stimulating each acupoint for 1 minute, repeated every 4 hours. The treatment was administered once daily, beginning 2 days prior to surgery and continuing until 3 days after surgery.
   3. **Control group**

Standard ERAS perioperative management (3.1).

**
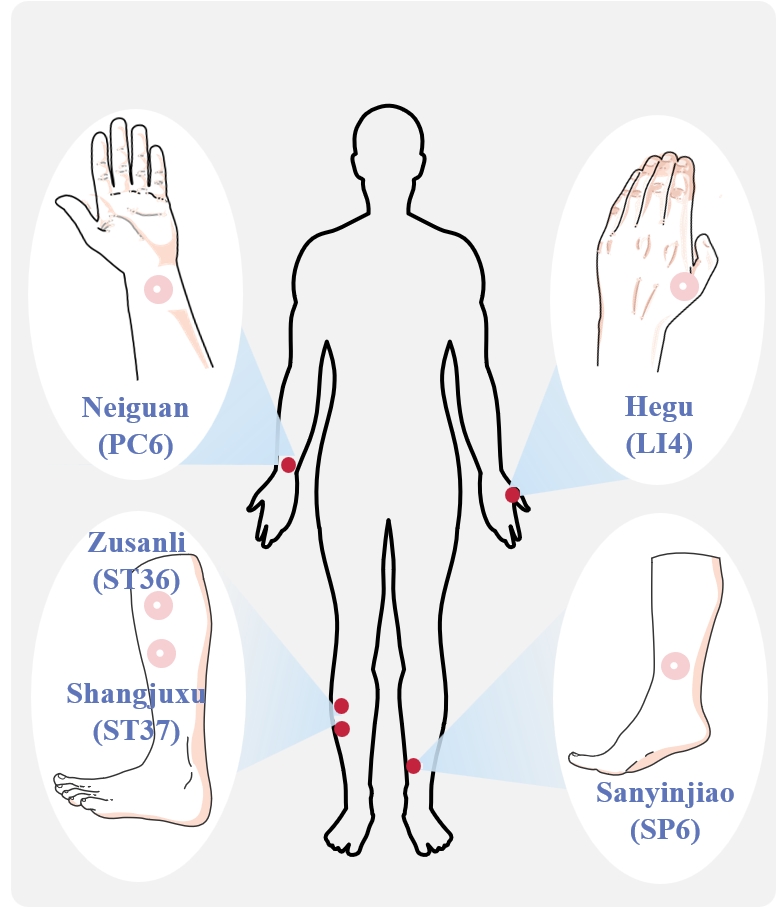
**

Figure 2 Locations of acupoints

1. **Outcome measurement**
   1. **Primary outcomes**
      1. **Time to bowel sound recovery:** The time from the end of the surgery to the restoration of bowel sounds to their preoperative normal state, measured in hours.
      2. **Time to first flatus:** The time from the end of the surgery to the first flatus, measured in hours.
   2. **Secondary outcomes**
      1. **Time to first defecation:** The time from the end of the surgery to the first defecation, measured in hours.
      2. **Time to removal of nasogastric tube:** The time from the end of the surgery until the determination that the nasogastric tube can be removed, measured in hours.
      3. **Time to removal of intra-abdominal drains:** The time from the end of the surgery until the determination that the drains can be removed, measured in hours.
      4. **Postoperative pain score:** Postoperatively, the Numerical Rating Scale (NRS) was employed to assess the pain status of two patient groups on the postoperative day 1 to 3. The NRS categorizes pain into 11 levels: 0 points: no pain; 1-3 points: mild pain; 4-6 points: moderate pain; 7-10 points: severe pain. Patients could self-assess their pain levels based on the corresponding numerical rating.
      5. **Postoperative nausea and vomiting score:** Postoperatively, the Visual Analogue Scale (VAS) was used to assess nausea and vomiting conditions of two patient groups on the postoperative day 1 to 3: utilizing a movable ruler approximately 10 cm long, marked with 10 graduations, with “0” at one end and “10” at the other end. A score of 0 indicates no nausea or vomiting, while 10 represents the most severe nausea or vomiting, with intermediate positions indicating different degrees of nausea or vomiting. Patients were instructed to mark the position on the ruler that represented their level of nausea or vomiting, indicating the severity of their condition.
      6. **Postoperative abdominal distention score:** Postoperatively, the Gastrointestinal Symptom Rating Scale (GSRS) was utilized to evaluate the abdominal distention condition of two patient groups on the postoperative day 1 to 3: GSRS categorizes abdominal distention into 7 levels, with 1 point denoting no discomfort, 2-3 points indicating mild abdominal distention, 4-5 points representing moderate abdominal distention, and 6-7 points indicating severe abdominal distention. Patients could self-assess their abdominal distention levels based on the corresponding numerical scale.
      7. **Postoperative hospital stay:** The duration from the end of the surgery until the patient's discharge, measured in days.
      8. **Postoperative complications:** Early complications occurring during the hospitalization period after subtotal or partial gastrectomy in gastric cancer patients include infection, bleeding, delayed gastric emptying, anastomotic leakage, etc.
      9. **Safety evaluation:** Acupuncture safety assessment includes dizziness from needling, retained or broken needles, intolerable needle pain (NRS ≥ 7), localized hematoma, and other discomforts post-acupuncture treatment (referring to sustained symptoms such as nausea, vomiting, dizziness, pain, palpitations, and loss of appetite persisting for at least 1 hour after acupuncture treatment); and unforeseen adverse events.
      10. **Overall response rate:** Based on the Rome IV criteria, assessment is conducted at 72 hours postoperatively. The sum of Complete response, Marked response, and Moderate response represents the overall response rate.
          1. Complete response: Normal restoration of flatus and defecation within 24 hours postoperatively, complete bowel sounds heard upon auscultation at 4-5 times/minute, no abdominal distension, only slight pain occurring during coughing, no nausea, no vomiting.
          2. Marked response: Flatus or defecation within 24-48 hours postoperatively, weaker bowel sounds at 2-3 times/minute, pain occurring during deep breathing, slight nausea without vomiting.
          3. Moderate response: Flatus or defecation within 48-72 hours postoperatively, abnormal bowel sounds at 1-2 times/minute, tolerable abdominal distension and pain at rest, significant nausea without emesis.
          4. No response: No flatus or defecation beyond 72 hours postoperatively, disappearance or extreme weakness of bowel sounds, no significant improvement or worsening of symptoms before and after treatment, obvious abdominal distension and pain, accompanied by nausea and vomiting.
2. **Ethical Principle**

This study protocol has been approved by ethics committees of the Affiliated Hospital of Guizhou Medical University (Approval No. 2023[6609]). This study conforms to the Declaration of Helsinki principles. Patient enrollment won't start until the Institution Review Board (IRB) approves the trial protocol, but everything should happen following registration.

1. **Statistical Analysis**

Statistical analyses were performed using SPSS 26.0 and R 4.1.0 software. Descriptive statistics were used to summarize the characteristics of patients in each group. For continuous variables, normality was assessed using the Shapiro-Wilk test. Data conforming to a normal distribution were presented as mean ± standard deviation (SD), and between-group comparisons were conducted using the independent samples t-test. Non-normally distributed data were reported as median [interquartile range (IQR)], and compared using the Mann-Whitney U test. The Hodges-Lehmann estimator was applied to compute the median differences and associated 95% confidence intervals (CIs). Categorical variables were compared using the chi-square (χ²) test or Fisher’s exact test, as appropriate. For variables with repeated measures and a normal distribution, analysis of variance (ANOVA) was used, followed by least significant difference (LSD) post hoc tests for pairwise comparisons at different time points. For non-normally distributed repeated measures, the Scheirer-Ray-Hare test was applied. A two-sided *P* value < 0.05 was considered statistically significant.

1. **Revision history**
2. The time to first bowel sound was changed as time to bowel sound recovery, because the former is difficult to monitor, the latter can better assess its efficacy.
3. The pain scoring standard was changed from VAS to NRS, because NRS is a specialized pain assessment scale.
4. The evaluation of nausea and vomiting was changed from subjective description to VAS.
5. The scoring standard for abdominal distension was adjusted from the criteria based on the Guidelines for Clinical Research of New Chinese Medicine to GSRS.
6. The interval time for thumb-tack needle was modified from 6 hours to 4 hours for better results, according to the advice of reviewers.
7. We ultimately decided not to use physiological and biochemical test data as evaluation criteria for this trial because earlier trials showed no significant difference between the two groups, and repeated blood tests led to some patient complaints.
8. **Funding**

This study was supported by the Guizhou Administration of Traditional Chinese Medicine of China (Grant No. QZYY-2024-061).

**References**

1.Jeddi F, Soozangar N, Sadeghi MR, et al; Nrf2 overexpression is associated with P-glycoprotein upregulation in gastric cancer. Biomedicine & pharmacotherapy 2018 Jan; 97:286-292.

2.Hyuna Sung, Jacques Ferlay, Rebecca L Siegel, et al; Global Cancer Statistics 2020: GLOBOCAN Estimates of Incidence and Mortality Worldwide for 36 Cancers in 185 Countries. CA: a cancer journal for clinicians 2021 05;71(3):209-249.

3.Tian Tian, Ling Xiao, Jiangbo Du, et al; Polymorphisms in CARS are associated with gastric cancer risk: a two-stage case-control study in the Chinese population. Gastric cancer: official journal of the International Gastric Cancer Association and the Japanese Gastric Cancer Association 2017 Nov;20(6):940-947.

4.Gastric cancer diagnosis and treatment specification (2018 edition). Chinese Journal of Digestion and Medical Imageology (Electronic Edition), 2019, 9(03): 118-144.

5.Expert consensus on enhanced recovery after gastrectomy for gastric cancer (2016 edition). Chinese Journal of Digestive Surgery, 2017, 16(1): 14-18.

6.H Kehlet; Multimodal approach to control postoperative pathophysiology and rehabilitation. British journal of anesthesia 1997 May;78(5):606-17.

7.Kehlet H, Joshi GP. Enhanced Recovery After Surgery: Current Controversies and Concerns. Anesth Analg. 2017, 125(6): 2154-2155.

8.Han Xudong; Wu Zhenzhen; Li Fang, et al. Effect of Postoperative Recovery of Gastrointestinal Function of Patients Undergoing Laparoscopic Hysterectomy in ERAS Procedure Combined with Acupoint Application and Traditional Chinese Herbs Foot Bath. Journal of Ningxia Medical University, 2021, 43(01): 48-51.

9.Xue Weidong, Li Hailong, Zhang Xianfeng. The effect of acupuncture anesthesia combined with remifentanil in the anesthesia of clavicle fracture surgery. Practical Clinical Journal of Integrated Traditional Chinese and Western Medicine, 2014, 14(6): 50-51.

10.Lan Xiaofang, Shu Jianzhong. Clinical observation of traditional Chinese medicine and oxygen intervention on preoperative anxiety in patients undergoing cerebral angiography. Yunnan Journal of Traditional Chinese Medicine and Materia Medica, 2019, 40(2): 45-47.

11.Guan Junjie, Wang Jingqiu, Zhou Weikang. Research progress of enhanced recovery after surgery combined with traditional Chinese medical. Chinese Journal of Acupuncture and Moxibustion, 202, 10(01):31-34.

12.Chen Lin, Chen Yajin, Dong Hailong. Chinese Expert Consensus and Pathway Management Guidelines on Enhanced Recovery After Surgery (2018 Edition). Chinese Journal of Practical Surgery, 2018, 38(01) :1-20.

13.Zhishun Liu, Shiyan Yan, Jiani Wu, et al; Acupuncture for Chronic Severe Functional Constipation: A Randomized Trial. Annals of internal medicine 2016 Dec 06; 165(11): 761-769.

14.Fan Yu Xiang. Clinical study of electro-acupuncture promoting early gastrointestinal function recovery after laparoscopic gastric cancer surgery. Nanjing University of Traditional Chinese Medicine, 2022
